# Development and validation of a new model for the early diagnosis of tuberculous meningitis in adults based on simple clinical and laboratory parameters

**DOI:** 10.1186/s12879-023-08922-5

**Published:** 2023-12-21

**Authors:** Qiang Liu, Meiling Cao, Na Shao, Yixin Qin, Lu Liu, Qing Zhang, Xiao Yang

**Affiliations:** 1 Department of Neurology, General Hospital of Ningxia Medical University, Ningxia Key Laboratory of Cerebrocranial Diseases, Incubation Base of National Key Laboratory, Yinchuan, 750004 Ningxia Province China; 2 Graduate College of Ningxia Medical University, Yinchuan, 750004 Ningxia Province China; 3 Department of Internal Medicine, The Inner Mongolia Autonomous Region, The People’s Hospital of Wushen Banner, Erdos, 017000 China; 4 Department of Neurology, The First People’s Hospital of Yinchuan, Yinchuan, 750004 Ningxia Province China

**Keywords:** Central nervous system infection, Tuberculous meningitis, Diagnostic model

## Abstract

**Background:**

The differential diagnosis between tuberculous meningitis (TBM) and viral meningitis (VM) or bacterial meningitis (BM) remains challenging in clinical practice, particularly in resource-limited settings. This study aimed to establish a diagnostic model that can accurately and early distinguish TBM from both VM and BM in adults based on simple clinical and laboratory parameters.

**Methods:**

Patients diagnosed with TBM or non-TBM (VM or BM) between January 2012 and October 2021 were retrospectively enrolled from the General Hospital (derivation cohort) and Branch Hospital (validation cohort) of Ningxia Medical University. Demographic characteristics, clinical symptoms, concomitant diseases, and cerebrospinal fluid (CSF) parameters were collated. Univariable logistic analysis was performed in the derivation cohort to identify significant variables (*P* < 0.05). A multivariable logistic regression model was constructed using these variables. We verified the performance including discrimination, calibration, and applicability of the model in both derivation and validation cohorts.

**Results:**

A total of 222 patients (70 TBM and 152 non-TBM [75 BM and 77 VM]) and 100 patients (32 TBM and 68 non-TBM [31 BM and 37 VM]) were enrolled as derivation and validation cohorts, respectively. The multivariable logistic regression model showed that disturbance of consciousness for > 5 days, weight loss > 5% of the original weight within 6 months, CSF lymphocyte ratio > 50%, CSF glucose concentration < 2.2 mmol/L, and secondary cerebral infarction were independently correlated with the diagnosis of TBM (*P* < 0.05). The nomogram model showed excellent discrimination (area under the curve 0.959 vs. 0.962) and great calibration (*P*-value in the Hosmer–Lemeshow test 0.128 vs. 0.863) in both derivation and validation cohorts. Clinical decision curve analysis showed that the model had good applicability in clinical practice and may benefit the entire population.

**Conclusions:**

This multivariable diagnostic model may help clinicians in the early discrimination of TBM from VM and BM in adults based on simple clinical and laboratory parameters.

## Background

Tuberculosis is caused by an infection with *Mycobacterium tuberculosis* and is one of the most prevalent infections in the world, with an estimated 2–3 billion individuals infected worldwide [1]. Tuberculous meningitis (TBM) is the most severe form of extrapulmonary tuberculosis [2] and leads to exceptionally high mortality and morbidity, largely due to difficulties in early diagnosis and treatment initiation [3–5], especially in resource-limited settings. Although the detection of pathogenic microorganisms is a reliable basis for TBM diagnosis, it is not only restricted by unsatisfactory sensitivity but also time-consuming [6, 7]. In recent years, emerging molecular biology detection techniques, such as the Xpert MTB/RIF assay [8, 9], have provided better diagnostic means. Although advanced molecular biology techniques have enabled great progress in the diagnosis of TBM, they have not been widely used in resource-limited high-risk areas for TBM owing to the high costs of these techniques. Therefore, simple, economical, and practical techniques that can be implemented effectively in resource-limited settings must be explored.

This study aimed to develop and validate a diagnostic score that can accurately predict TBM at an early stage by comparing TBM with other clinically common meningitis types (viral meningitis [VM] and bacterial meningitis [BM]) as they may mimic each other due to the nonspecific clinical presentations of these three meningitis types.

## Methods

### Derivation and validation cohorts

From January 2012 to October 2021, patients (≥ 18 years of age) with a diagnosis of TBM, BM, or VM in the General Hospital (derivation cohort) and Branch Hospital (validation cohort) of Ningxia Medical University were retrospectively reviewed. We strictly followed the protocol and guidance set out according to the TRIPOD10 (Transparent Reporting of a Multivariable Prediction Model for Individual Prognosis or Diagnosis) [10] statement for reporting multivariable prediction model development and validation.

Individuals were enrolled in the TBM group when they had been diagnosed with highly probable or definite TBM according to the Marais uniform TBM case definition [11]. A definite diagnosis of TBM was made when one or more of the following criteria were met: 1) positive staining for acid-fast bacilli in the cerebrospinal fluid (CSF); 2) *M. tuberculosis* was cultured from the CSF; or 3) a *M. tuberculosis* nucleic acid amplification test was positive in the CSF from a patient who presented with symptoms or signs suggestive of meningitis. Highly probable TBM was determined using a diagnostic scoring system requiring the presence of symptoms or signs indicative of meningitis plus additional clinical, CSF, or imaging criteria, with the exclusion of the most likely alternative diagnoses. The diagnostic criteria for VM [12] were as follows: 1) viruses were isolated or specific antibodies were identified from CSF, or 2) the patients presented with meningitis symptoms, and there was no evidence of additional pathogenic microorganisms, the antiviral treatment was effective. A BM diagnosis was required to fulfil the following criteria [13]: 1) a pathogenic bacterium was isolated or cultured from the CSF or 2) the patients presented with meningitis symptoms and the following conditions were simultaneously met: 2a) the CSF white blood cell count was > 1000 × 10^6^ cells/L and 2b) there was no evidence of additional pathogenic microorganisms, and the antibacterial treatment was effective.

Patients with any of the following conditions were excluded from the study: 1) age < 18 years, 2) insufficient data, 3) mixed infection, 4) anti-tuberculosis treatment before admission, and 5) symptoms were attributed to cerebral trauma or neurosurgery.

### Candidate predictor variables

We identified candidate predictor variables for inclusion in our model from literature reports and clinical experience, then collated information on these potential predictor variables during the index hospitalisation for meningitis. Briefly, we collected information on demographic data (age at onset, sex), duration (interval from symptom onset to hospital admission), clinical symptoms (headache, fever, vomiting, nuchal rigidity, convulsions, disturbance of consciousness, persistent cough, weight loss, cranial nerve palsy, and focal neurologic deficit), concomitant diseases (cerebral infarction, hydrocephalus, extracranial tuberculosis, and hyponatremia), and CSF parameters (intracranial pressure, cell count, lymphocyte percentage, protein, glucose, and chloride) in the first lumbar puncture.

### Model construction and validation

First, predictive factors independently related to TBM diagnosis were screened in the derivation cohort by univariable regression analysis. Then, logistic multivariable regression analysis was performed, and the TBM diagnosis model was established using stepwise backward regression. Subsequently, the model was evaluated and validated under the aspects of discrimination, calibration, and clinical applicability. The area under the receiver operating characteristic (ROC) curve (AUC) was used to evaluate differentiation. The Hosmer–Lemeshow goodness-of-fit test and calibration curve were used to evaluate calibration, and decision curve analysis was used to evaluate clinical applicability. Finally, we generated a nomogram to visualise the model, thereby making it simple and intuitive for practical applications.

### Statistical analysis

Demographic characteristics, clinical symptoms, concomitant diseases, and CSF parameters of patients who fulfilled the diagnostic criteria for TBM and non-TBM (BM and VM) were compared. Categorical variables are expressed as numbers (percentages) and compared using the chi-square test. All statistical tests were two-sided, and statistical significance was set at *P* < 0.05. Variables with *P* < 0.05 were entered stepwise into logistic regression analysis using the backward conditional method. Before introducing candidate variables into the logistic regression analysis, all variables were dichotomised based on clinical experience. Multivariable logistic regression was used to create the diagnostic model. The regression coefficients of the model were regarded as weights for the respective variables, and the scores for each patient were calculated. Data were analysed using Stata version 15.0.

## Results

### Characteristics of the derivation and external validation cohorts

**Fig. 1:**
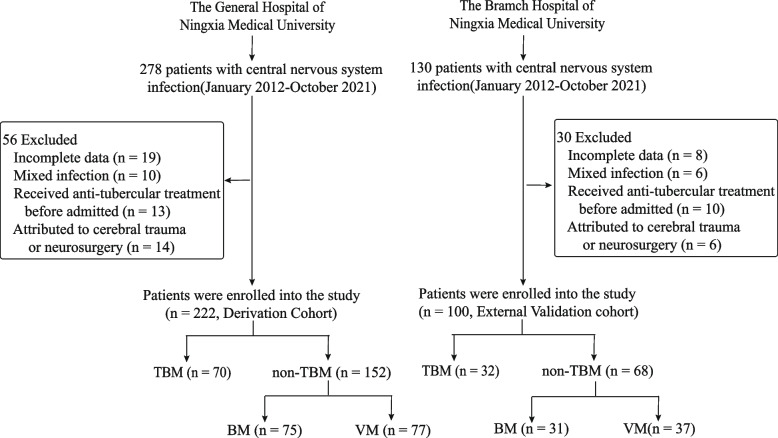
Flowchart of patient enrolment into the derivation and validation cohorts. BM: bacterial meningitis, TBM: tuberculous meningitis, VM: viral meningitis

In total, 322 patients were enrolled in either the derivation (*n* = 222) or external validation (*n* = 100) cohort. The details of the enrolment process are shown in the flowchart (Fig. 1). The 23 relevant demographic characteristics, clinical symptoms, concomitant diseases, and CSF parameters of the two cohorts are summarised in Table 1. Headache and fever were the most common symptoms in both cohorts. Compared with patients in the derivation cohort, patients in the external validation cohort were more likely to present with cranial nerve palsies (*P* = 0.041) and less prone to developing headaches (*P* = 0.005). The remaining 21 variables were not significantly different between the two cohorts (*P* > 0.05).

**Table 1:** Demographic and clinical characteristics of the derivation and validation cohorts

Variable	Derivation cohort (*n* = 222)	Validation cohort (*n* = 100)	*P*-value
Age at onset ≤ 60 years, n (%)	195 (87.8)	82 (82.0)	0.162
Male sex, n (%)	129 (58.1)	65 (65.0)	0.242
Duration of symptoms > 5 days, n (%)	113 (50.9)	54 (54.0)	0.607
Symptoms			
Headache, n (%)	215 (96.8)	89 (89.0)	0.005
Fever, n (%)	202 (91.0)	86 (86.0)	0.177
Vomiting, n (%)	152 (68.5)	60 (60.0)	0.138
Nuchal rigidity, n (%)	145 (65.3)	66 (60.0)	0.905
Convulsions, n (%)	29 (13.1)	12 (12.0)	0.791
Disturbance of consciousness, n (%)	66 (29.7)	37 (37.0)	0.196
Persistent cough > 2 weeks, n (%)	7 (3.2)	3 (3.0)	> 0.99
Weight loss > 5% of the original weight within 6 months, n (%)	24 (10.8)	9 (9.0)	0.620
Cranial nerve palsies, n (%)	4 (1.8)	7 (7.0)	0.041
Focal neurologic deficit, n (%)	10 (4.5)	2 (2.0)	0.435
Concomitant diseases			
Cerebral infarction, n (%)	11 (5.0)	8 (8.0)	0.283
Hydrocephalus, n (%)	13 (5.9)	11 (11.0)	0.104
Extracranial tuberculosis, n (%)	37 (16.7)	22 (22.0)	0.252
Hyponatremia < 135 mmol/L, n (%)	71 (32.0)	39 (39.0)	0.219
CSF alterations			
Intracranial pressure (> 180 mmH_2_O), n (%)	172 (77.5)	69 (69.0)	0.105
CSF cell count (> 500 × 10^6^ /L), n (%)	64 (28.8)	29 (29.0)	0.975
CSF lymphocyte percentage (> 50%), n (%)	137 (61.7)	61 (61.0)	0.903
CSF protein concentration (> 1 g/L), n (%)	153 (68.9)	72 (72.0)	0.577
CSF glucose concentration (< 2.2 mmol/L), n (%)	103 (46.4)	49 (49.0)	0.665
CSF chloride concentration (< 120 mmol/L), n (%)	151 (68.0)	60 (60.0)	0.161

*CSF* cerebrospinal fluid

### Identification of variables to differentiate TBM from non-TBM

Univariable regression analysis was performed on 23 variables from the two groups (70 patients in the TBM group and 152 patients in the non-TBM group) in the derivation cohort to identify variables of diagnostic significance (Table 2). The results demonstrated many significant differences between the TBM group and non-TBM group, including age at onset (≤ 60 years), sex, duration (> 5 days), disturbance of consciousness, persistent cough (> 2 weeks), weight loss (> 5% of the original weight within 6 months), focal neurologic deficit, cerebral infarction, hydrocephalus, extracranial tuberculosis, hyponatraemia < 135 mmol/L, intracranial pressure (> 180 mmH_2_O), CSF cell count (> 500 × 10^6^ /L), CSF lymphocyte percentage (> 50%), CSF protein concentration (> 1 g/L), CSF glucose concentration (< 2.2 mmol/L), and CSF chloride concentration (< 120 mmol/L). The full details of the results of this analysis are presented in Table 2.

**Table 2:** Univariable analysis of predictors distinguishing between TBM and non-TBM

Variable	TBM (*n* = 70)	Non-TBM (*n* = 152)	*P*-value	OR (95% CI)
Age at onset ≤ 60 years, n (%)	56 (80.0)	139 (91.4)	0.015	0.88 (0.77–0.99)
Male sex, n (%)	31 (44.3)	98 (64.5)	0.005	0.69 (0.52–0.92)
Duration of symptoms > 5 days, n (%)	63 (90.0)	50 (32.9)	< 0.001	2.74 (2.15–3.48)
Symptoms				
Headache, n (%)	65 (92.9)	150 (98.7)	0.058	0.94 (0.88–1.01)
Fever, n (%)	63 (90.0)	139 (91.4)	0.726	0.98 (0.90–1.08)
Vomiting, n (%)	47 (67.1)	105 (60.1)	0.773	1.06 (0.71–1.60)
Nuchal rigidity, n (%)	52 (74.3)	93 (61.2)	0.057	1.21 (1.01–1.46)
Convulsions, n (%)	10 (14.3)	19 (12.5)	0.714	1.14 (0.56–2.33)
Disturbance of consciousness, n (%)	37 (52.9)	29 (19.1)	< 0.001	2.77 (1.87–4.11)
Persistent cough > 2 weeks, n (%)	6 (8.6)	1 (0.7)	0.006	13.03 (1.50–106.18)
Weight loss > 5% of the original weight within 6 months, n (%)	20 (28.6)	4 (2.6)	< 0.001	10.86 (3.86–30.58)
Cranial nerve palsies, n (%)	1 (1.4)	3 (2.0)	> 0.99	0.72 (0.08–6.84)
Focal neurologic deficit, n (%)	61 (87.1)	151 (99.3)	< 0.001	0.88 (0.80–0.96)
Concomitant diseases				
Cerebral infarction, n (%)	10 (14.3)	1 (0.7)	< 0.001	21.7 (2.84–166.34)
Hydrocephalus, n (%)	13 (18.6)	0 (0.0)	< 0.001	-
Extracranial tuberculosis, n (%)	37 (52.9)	0 (0.0)	< 0.001	-
Hyponatremia < 135 mmol/L, n (%)	43 (61.4)	28 (18.4)	< 0.001	3.34 (2.28–4.89)
CSF alterations				
Intracranial pressure (> 180 mmH_2_O), n (%)	61 (87.1)	111 (73.0)	0.019	1.19 (1.05–1.36)
CSF cell count (> 500 × 10^6^ /L), n (%)	3 (4.3)	61 (40.1)	< 0.001	0.11 (0.04–0.33)
CSF lymphocyte percentage (> 50%), n (%)	57 (81.4)	80 (52.6)	< 0.001	1.55 (1.28–1.87)
CSF protein concentration (> 1 g/L), n (%)	61 (87.1)	92 (60.5)	< 0.001	1.44 (1.23–1.68)
CSF glucose concentration (< 2.2 mmol/L), n (%)	58 (82.9)	45 (29.6)	< 0.001	2.80 (2.14–3.66)
CSF chloride concentration (< 120 mmol/L), n (%)	66 (94.3)	85 (55.9)	< 0.001	1.69 (1.45–1.96)

*CI* confidence interval, *CSF* cerebrospinal fluid, *OR* odds ratio, *TBM* tuberculous meningitis, non-*TBM* viral or bacterial meningitis

### Establishing the diagnostic model discriminating TBM from non-TBM

Significant variables in the univariable analysis were subsequently included in the multivariable logistic regression analysis. Six variables (duration, disturbance of consciousness, weight loss, cerebral infarction, CSF lymphocyte percentage, and CSF glucose concentration) were found to be independently associated with TBM according to stepwise backward logistic regression analysis (Table 3). A logistic regression equation for determining the joint probability of the six variables was then obtained to predict the probability of a TBM diagnosis. The score for each predictor was determined using the odds ratio (OR) in the logistic regression equation.

**Table 3:** Results of multivariable logistic regression analysis in the derivation cohort

Predictors	β	S.E	Wald χ^2^	OR	95% CI	*P*-value
Duration of symptoms (> 5 days)	3.21	0.67	23.29	24.82	6.74–91.48	< 0.001
Disturbance of consciousness	1.79	0.67	7.08	5.97	1.60–22.27	0.008
Weight loss (> 5% of the original weight within 6 months)	3.50	0.98	12.71	33.17	4.84–227.33	< 0.001
Cerebral infarction	2.86	1.25	5.22	17.51	1.50–204.18	0.022
CSF lymphocyte percentage (> 50%)	2.73	0.71	14.71	15.28	3.79–61.52	< 0.001
CSF glucose concentration (< 2.2 mmol/L)	3.45	0.66	27.20	31.44	8.61–114.89	< 0.001

*CI* confidence interval, *CSF* cerebrospinal fluid, *OR* odds ratio

### Validation and evaluation of the established diagnostic model by an independent cohort

**Fig. 2:**
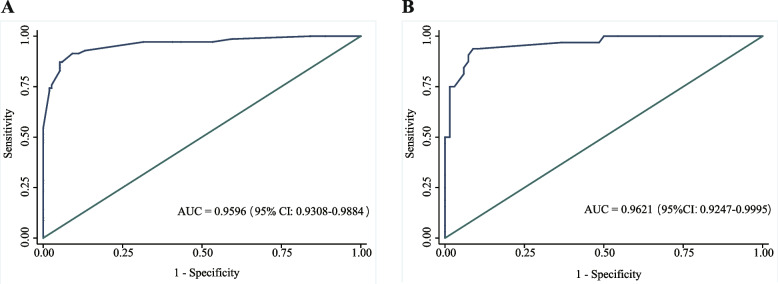
The ROC curves of the TBM diagnostic model in the derivation (**A**) and validation (**B**) cohorts. The ROC curves show the specificity and sensitivity of predicting TBM in the derivation and validation cohorts based on the model output. These values indicate the good discrimination ability of the diagnostic model. AUC: area under the curve, CI: confidence interval, ROC: receiver operating characteristic, TBM: tuberculous meningitis

**Fig. 3:**
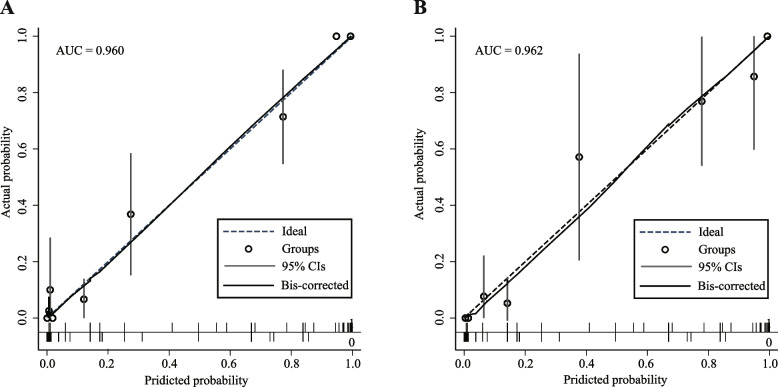
Calibration curves for the nomogram model in the derivation (**A**) and validation (**B**) cohorts. The x-axis represents the forecasted TBM risk, whereas the actual diagnosed TBM is shown on the y-axis. For each subsequent decile, the observed TBM rate in the cohort was plotted against the model prediction (black circle, average; grey line, 95% CI). The diagonal dotted line represents the ideal model with perfect prediction ability, and the solid line (bias-corrected line) represents the real performance of the nomogram. The closer the fit to the diagonal dotted line, the better the prediction ability of the nomogram. The nomogram model was excellently calibrated in both derivation and validation cohorts. AUC: area under the curve, CI: confidence interval, TBM: tuberculous meningitis

**Fig. 4:**
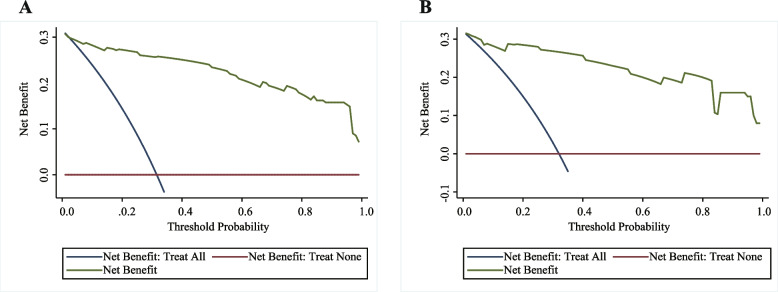
Clinical usefulness measured by decision curve analysis. The y-axis represents the net benefit. Net benefit is calculated across a range of threshold probabilities, defined as the minimum probability of disease at which further intervention would be warranted, as net benefit = sensitivity × prevalence – (1 – specificity) × (1 – prevalence) × w where w is the odds at the threshold probability. The green line represents the predicted line for a diagnostic model of tuberculous meningitis at a threshold probability ranging from 0 to 1.0. The nomogram adds net benefits compared to the treat-none (blue) and treat-all (pink) conditions in the decision curve

**Fig. 5:**
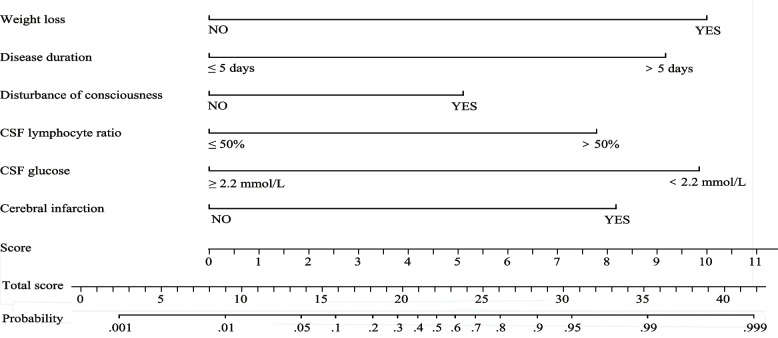
Nomogram for predicting the probability of TBM. Individual patient values were based on each variable axis of the nomogram, and the number of points obtained for each variable was determined using a line drawn downward. The sum of the points is located on the total score axis, which corresponds on the line below to the probability of TBM. CSF: cerebrospinal fluid, TBM: tuberculous meningitis

The ROC curves and AUC values are shown in Fig. 2. The AUC value was 0.9596 (95% confidence interval [CI] 0.9308–0.9884) in the derivation cohort and 0.9621 (95% CI 0.9247–0.9995) in the validation cohort. The optimal cut-off value in the derivation cohort with a sensitivity of 91.4% and a specificity of 90.8% was set at 0.217. A similar result was obtained in the validation cohort; the optimal cut-off value with a sensitivity of 93.8% and a specificity of 91.2% was set at 0.213. These results indicate that the model has a reasonably good discrimination ability for separating TBM from non-TBM. Calibration refers to the accuracy of a model in predicting the probability of an event. Model calibration evaluates the degree to which the model predictions fit the observed data across different stratifications. The Hosmer–Lemeshow goodness-of-fit test indicated that the predictive performance of the model was excellent (Fig. 3). Good agreement was observed between the observed and predicted probabilities of TBM (*P* = 0.1278 in the derivation cohort and *P* = 0.8634 in the validation cohort). The practicability of this model was analysed using a decision curve, as shown in Fig. 4. The decision curve showed that the models presented net benefits over the entire range of threshold probabilities 0–1.0, with better performance than the two extreme conditions (treat-none and treat-all). Thus, decision curve analysis demonstrated that this model has high clinical usefulness. Multivariable regression models are widely used in medical literature for the purpose of diagnosis or prediction. Conventionally, the adequacy of these models is assessed using metrics of diagnostic performances such as sensitivity and specificity, which fail to account for clinical utility of a specific model. Decision curve analysis is a widely used method to measure this utility. In this framework, a clinical judgment of the relative value of benefits (treating a true positive case) and harms (treating a false positive case) associated with prediction models is made. As such, the preferences of patients or policy-makers are accounted for by using a metric called threshold probability. A decision analytic measure called net benefit is then calculated for each possible threshold probability, which puts benefits and harms on the same scale. In brief, decision curve analysis calculates a clinical “net benefit” for prediction models in comparison to default strategies of treating all or no patients. This function also allows to calculate the different kinds of net benefits (treat all, treat none, and different threshold probability. To visualise the model, we constructed a nomogram to predict the probability of TBM in patients with meningitis based on six variables (duration, disturbance of consciousness, weight loss, cerebral infarction, CSF lymphocyte percentage, and CSF glucose concentration). The higher the total score, the higher the risk of TBM (Fig. 5).

## Discussion

Owing to the often atypical clinical presentation of TBM and the low bacterial load in the CSF, early diagnosis of TBM can be challenging, frequently leading to misdiagnoses as purulent, viral, or cryptococcal meningitis. TBM diagnosis is primarily based on CSF smear detection or cultures of isolated *M. tuberculosis*. However, both strategies have limited clinical value for the early diagnosis of TBM owing to their low positive rates [14, 15]. Although Xpert MTB/RIF has improved the accuracy of TBM diagnosis and shortened the time required to initiate appropriate treatment [8, 9], this technique is not widely available, particularly in resource-limited settings due to the requirement for specialized equipment and high costs. Potential proteome [16], metabolome [17], and transcriptome [18] biomarkers have been identified for TBM diagnosis in recent years; however, these markers have not been sufficiently verified. Therefore, clinicians still depend on medical history, clinical presentation, and simple laboratory parameters to aid TBM diagnosis.

In the past few years, an increasing number of studies have attempted to distinguish TBM from non-TBM using clinical prediction models [19–23] which were constructed by integrating clinical presentation, laboratory tests, and imaging examinations. A recent study [24] successfully uncovered and established a diagnostic model based on a combination of the TB-specific antigen/phytohemagglutinin (TBAg/PHA) ratio, CSF chloride concentration, CSF nucleated cell count, and CSF lymphocyte proportion, with excellent utility in distinguishing TBM from BM. However, this model has several limitations. First, the prior study included only patients with microbiologically confirmed TBM; in clinical practice, the diagnosis of possible TBM is often critical [11, 25]. Second, its clinical value is limited because it could not discriminate between TBM, VM, and BM simultaneously, as the model only used the BM group as a control. More importantly, the predictor TBAg/PHA is laborious and requires specialised equipment that is unavailable in economically underdeveloped and geographically remote areas. Other TBM diagnostic models have similar limitations. For example, they were only compared with VM [21, 26] or ignored the cost-effectiveness of model applications in actual clinical environments [24, 25]. However, the development and performance of an accurate prediction model will depend to a large extent on the population studied and the factors involved in developing and testing the prediction model.

In the current study, we developed and validated a new diagnostic scoring system by simultaneously comparing 23 factors (including clinical symptoms, concomitant diseases, and CSF parameters) of TBM with those of VM and BM in adult patients. Univariable analysis of admission variables suggested a set of potentially discriminative clinical and laboratory features (Table 2). Multivariable logistic regression analysis defined six characteristics independently predictive of the distinction between TBM and non-TBM: duration of symptoms > 5 days, weight loss > 5% of the original weight within 6 months, disturbance of consciousness, CSF lymphocyte percentage > 50%, CSF glucose concentration < 2.2 mmol/L, and secondary cerebral infarction (Table 3). Although Marais et al. [11] have used these parameters in the clinical practice for the diagnosis of tuberculous meningitis, we have taken a different approach. We have developed a predictive model for the diagnosis of tuberculous meningitis, incorporating these valuable parameters as variables. Instead of relying on a single independent parameter, our model utilizes multiple variables, which may enhance the accuracy of the TBM diagnosis.

As in previous reports, we found that a symptom duration ≥ 5 days was an important predictor for TBM because patients with TBM do not suddenly present classic meningitis symptoms [11]. The pathogenesis of TBM mainly manifests as the accumulation of *M. tuberculosis* in the body. When host immunity is low, *M. tuberculosis* weakens the blood–brain and blood-CSF barriers through molecular biological mechanisms, and these processes require some time. This explains the longer course of TBM compared to those of other meningitis types [27]. Patients with TBM often manifest nonspecific symptoms, including fatigue, fever, and weight loss, prior to onset. Our results demonstrated that weight loss > 5% of the original weight within 6 months was the strongest significant predictor for the diagnosis of TBM, which can be attributed to tuberculosis bacteria consuming calories. Our study also suggested that disturbance of consciousness is an independent risk factor associated with TBM diagnosis. TBM is more likely to affect the brain parenchyma than VM or BM. Tuberculoma, brain abscess, and cerebral infarction are important causes of consciousness disturbances. In an observational study of patients newly diagnosed with TBM [28], neuroendocrine dysfunction occurred in half of the study population. This is likely due to the tendency of TBM to affect basal structures such as the pituitary gland, pituitary stalk, and hypothalamus. Exudates lead to oedema, perivascular infiltration, and subsequent microglial reactions. Hydrocephalus and cerebral oedema may also be the main mechanisms leading to disorders of consciousness. The incidence of cerebral infarction in patients with TBM ranges from 6 to 47%, and cerebral infarction is the main risk factor for disability and death from TBM [29, 30]. Our study showed that cerebral infarction was observed in 14.3% of patients with TBM, and multivariable regression analysis identified cerebral infarction as an independent risk factor associated with the diagnosis of TBM. However, the pathogenesis of TBM-associated cerebral infarction remains unclear. The intracerebral pathology of TBM is mediated by dysregulated inflammatory responses, which may involve thickening of the vessel intima, resulting in vessel stenosis or occlusion [31]; this is probably the main mechanism. Various other pathogenetic mechanisms have been suggested, including vasculitis, arterial thrombosis, and vascular proliferation [32, 33].

It is well-known that CSF laboratory data play a vital role in the diagnosis of meningitis. This study indicated that CSF-glucose content (< 2.2 mmol/L) has a strong independent association with TBM, which is in line with the manifestations of TBM in a previous report [34]. Notably, the results of previous studies [11, 19, 24, 35, 36] were inconsistent with regard to the classification of the dominant proportion of CSF cells in the diagnosis of TBM. The crucial element of our TBM scoring system is a predominance of CSF lymphocytes but not neutrophils. Meninges are a special type of membrane in the brain containing blood and lymphatic vessels that contain large numbers of lymphocytes. In TBM, the interaction of *M. tuberculosis* with meningeal epithelium and lymphocytes causes an inflammatory reaction. The pathogen enters the lymph nodes through the blood and lymphatic vessels, causing infiltration of inflammatory cells, leading to an inflammatory reaction in the meninges and an increase in the number of lymphocytes in the CSF [27, 37]. We speculate that the predominance of cell classification varies in different studies, which may be attributed to differences in the methods of classifying CSF cells, differences in the timing of CSF collection, and differences in the patient populations referenced [24, 36].

Overall, our TBM diagnostic model has been an improvement compared with those of previous studies and is particularly suitable for resource-limited settings. First, highly probable and definite TBM was included in our model according to international diagnostic criteria and expert consensus. This is more in line with clinical practice in comparison to most previous models which only included patients with definite TBM. Second, the predictors in our model were primarily based on demographic, clinical, and laboratory features found in the literature review and clinical experience. Remarkably, all are inexpensive, easy to obtain in clinical practice, highly feasible, and can be carried out widely in hospitals at all levels, which is instructive in clinical practice. Third, the present study conducted an external validation by collecting data from an independent cohort of another grade IIIA hospital, and the model was evaluated and validated based on differentiation, calibration, and clinical applicability. Both derivation and validation cohorts presented very high AUC values in their ROC curves (Fig. 2) and excellent calibration for the full model (Fig. 3). Moreover, clinical decision curve analysis demonstrated that most patients with TBM benefit from the diagnostic model (Fig. 4). In addition, our diagnostic model was visualised in the form of a nomogram which could be effectively applied to clinical decision making (Fig. 5).

### Limitations

Although we achieved satisfactory clinical outcomes, this study has several limitations. First, only a limited number of variables were included in the logistic regression analysis because this was a retrospective study, and some patients were excluded. Second, the sample size was relatively small, and the data were limited to the northwestern region of China. In the future, the sample size should be expanded to explore whether different hospitals in different regions can validate our model. Finally, non-TBM in this study cohort included only VM and BM. However, in clinical practice, meningitis can have a wider range of causes, including fungal infections and autoimmune diseases. This slightly affects the accuracy of the model in clinical applications.

## Conclusions

Our study established a novel diagnostic model based on a combination of six indicators with excellent utility in distinguishing TBM from non-TBM, particularly in settings with limited resources for pathogen detection and molecular biology techniques.

## Data Availability

The datasets generated and/or analysed during the current study are not publicly available but are available from the corresponding author upon reasonable request.

## Abbreviations

AUC: Area under the curve
BM: Bacterial meningitis
CI: Confidence interval
CSF: Cerebrospinal fluid
non-TBM: Non-tuberculous meningitis
OR: Odds ratio
ROC: Receiver operating characteristic
TBAg/PHA: TB-specific antigen/phytohemagglutinin
TBM: Tuberculous meningitis
VM: Viral meningitis
